# Sauerbruch, STARPAHC, and SARS: Historical Perspectives on Readiness and Barriers in Telemedicine

**DOI:** 10.1007/s10389-021-01513-1

**Published:** 2021-03-24

**Authors:** Doreen Reifegerste, Lorenz Harst, Lena Otto

**Affiliations:** 1 School of Public Health, Bielefeld University, Universitätsstrasse 25, 33615 Bielefeld, Germany; 2 Technische Universität Dresden, Dresden, Germany

**Keywords:** Barriers, History of Medicine, Pandemic, Readiness factors, Telemedicine, Telemedicine Community Readiness Model

## Abstract

**Aim:**

Telemedicine is a promising solution to extend traditional health care services. Even though mainly discussed during the past two decades, its roots go back into the past century and even further, considering the use of bonfires to warn other villages of diseases. Insights from historical cases can therefore be useful for the ongoing discussion regarding the successful implementation of telemedicine.

**Subject and Methods:**

We analyzed three historical telemedicine cases (varying regarding time and place) and extracted their success factors and barriers as well as assessed their maturity by using the Telemedicine Community Readiness Model (TCRM). Evidence-based categories of success factors and barriers as well as the TCRM’s dimensions were used as deductive categories to analyze the study material’s content.

**Results:**

The analysis showed that the readiness for telemedicine is higher when the technology is the only option to access health care services. In all three cases, core readiness played a central role. However, the health sector, existing technology, and finance were barriers present at all times, while during pandemics, some barriers are only temporarily removed, for example, by putting legal issues on hold. The analyzed cases were all on lower levels of maturity as they mainly represent pilot tests or exceptional circumstances.

**Conclusion:**

Results indicate the important core functions in telemedicine initiatives as well as the diversity of their circumstances. Insights from such historical meta-perspectives can, for example, help to strengthen the sustainability of the increased use of telemedicine during the COVID-19 pandemic and scale up current telemedicine projects.

## Introduction

Telemedicine is by no means an invention of the twenty-first century but has roots that go back thousands of years to the use of bonfires to warn other villages of diseases (Bashshur and Shannon 2009). In a broader sense, telemedicine can be understood as “the conveyance of health information using the best technology available” (Hurst 2016), always with the final means to bring care to those in need of it (Sood et al. 2007). With telemedicine being such a historical concept, looking into the past can provide important insights for its successful implementation in the present as well as the future.

Thus, this study pursues two objectives: The first aim is to scrutinize selective historical cases of telemedicine use against the background of present concepts of facilitators and barriers of telemedicine projects to understand the enablers and barriers of telemedicine (Harst et al. 2019a). The second aim is to define the maturity level of the historical cases based on the criteria of the Telemedicine Community Readiness Model (TCRM) (Care4Saxony 2020; Otto et al. 2020b), which is a generic concept of readiness for telemedicine in communities.

Our research is especially necessary as telemedical projects still seem to be very limited in their scaling-up—despite their potentials and promises (Huang et al. 2017). These potentials and promises have hardly changed within the past century. The concept of telemedicine is strongly connected to the hope of increasing access to care for otherwise underserved groups, e.g., in a rural context (Bashshur et al. 2000). However, research often focusses either solely on acceptance by end-users (Harst et al. 2019b), selected geographical locations and corresponding health care systems (Helitzer et al. 2003), or specific application types for different medical settings (Rogers et al. 2017). Thus, there is a lack of a holistic as well as a long-term assessment of telemedicine projects (Hastall et al. 2017). Here, historical cases using different technology can provide a long-term perspective often missing in current studies (Timpel and Harst 2020).

In the following, the term telemedicine is defined as health care delivery and/or medical education either between health professionals and patients or among the health professionals involved that overcome (geographical) distances by using information and communication technologies (Otto et al. 2020a). Following this definition, we look at historical projects that describe telemedicine initiatives adhering to this understanding.

To fulfill the aims stated above, we pose three research questions guiding this article:

- RQ1: What are the success factors of historical telemedicine projects?
- RQ2: What are the barriers to the successful implementation of historical telemedicine projects?
- RQ3: What readiness levels were reached by historical telemedicine projects?

To answer the three research questions, we apply existing categories of success factors and barriers for telemedicine initiatives and classify the three historical projects into an existing assessment tool, the TCRM.

## Background

The successful implementation and scale-up of telemedicine projects are influenced by several factors, which can either facilitate or prevent progress. Factors facilitating the implementation and scale-up of telemedicine are success factors often related to readiness. Readiness is thereby referred to as the “degree to which users, healthcare organizations, and the health system itself, are prepared to participate and succeed” (The Alliance for Building Capacity 2002) in/with telemedicine implementation. On the contrary, barriers resulting from the involvement of different people, processes, or objects can prevent successful implementation and the subsequent scale-up of telemedicine projects.

In the following, we summarize findings from prior work regarding success/readiness and barrier factors, which will afterward be checked for fulfillment in the selected historical cases. Additionally, the TCRM is introduced, which will also be applied to the historical cases.

### Success factors

To identify success factors supporting telemedicine implementation and scale-up, a review of existing telemedicine maturity and readiness models was undertaken because they are assumed to include such factors (Otto et al. 2019). Out of ten such models, 91 factors were extracted and clustered into ten categories of readiness.

One necessary precondition for telemedicine implementation is core readiness, describing a need combined with a dissatisfying status quo or a desire to change, while telemedicine is seen as a useful alternative (The Alliance for Building Capacity 2002). Furthermore, patients, health care providers, and the community need to be ready, as well as the health sector itself. Further categories are strategic and organizational, financial and legal, and technological readiness. To clarify what each category encompasses, they are illustrated with examples in Table 1.

**Table 1:** Examples of success factors for telemedicine implementation and scale-up (Otto et al. 2019)

Success factor category	Examples
Core readiness	Identification and prioritization of future needs and dissatisfaction with the status quo (Jennett et al. 2003)
Patient readiness	Patient needs as the basis for telemedicine; ability to use equipment (Jensen et al. 2015)
Provider readiness	Willingness of use (Khoja et al. 2007; van Dyk and Schutte 2012); qualification (van Dyk and Schutte 2012)
Community readiness	Positive underlying culture in the region (Jensen et al. 2015)
Health sector readiness	Trust between stakeholders (Jensen et al. 2015); awareness and support of information and communication technologies on the part of politicians and policymakers at an institutional level (Abera et al. 2014; Khoja et al. 2007)
Strategic readiness	Marketing and promotion strategy (iCOPS 2017; Jennett et al. 2003)
Organizational readiness	Organizational regulatory framework (Abera et al. 2014; Jennett et al. 2003)
Financial readiness	Funding (Abera et al. 2014; iCOPS 2017; Jensen et al. 2015), reimbursement policies (Jennett et al. 2003; Khoja et al. 2007)
Legal readiness	Legal and regulatory framework (Abera et al. 2014; Jensen et al. 2015; Khoja et al. 2007)
Technological readiness	Appropriate infrastructure (Jennett et al. 2003; Jensen et al. 2015; Khoja et al. 2007), interoperability of technology used (Broens et al. 2007; Sokolovich and Fera 2015)

### Barriers to telemedicine implementation

A systematic overview of implementation barriers for telemedicine initiatives, which summarized existing reviews, found 98 barriers for telemedicine implementation (Otto and Harst 2019). These barriers include 11 factors triggering the barriers: patients and health care providers, their culture and the patient’s disease, the health sector, standards/guidelines, legal framework, finance, organization, and methodology as well as the technology applied. Typical barriers of telemedical problems in current times are limited financial resources, the resistance of individual end-users (especially patients and health care providers), and lacking regional infrastructure (Otto and Harst 2019). The barrier categories are further illustrated in Table 2 by presenting examples per category.

**Table 2:** Examples of telemedicine implementation barriers (Otto and Harst 2019)

Barrier category	Examples
Patient	Resistance to change (Kruse et al. 2016; Saliba et al. 2012), low income (Hage et al. 2013; Jang-Jaccard et al. 2014)
Health care provider	Fear of loss of system/patient control (Jang-Jaccard et al. 2014; Saliba et al. 2012)
Culture	Culturally inappropriate communication (Jang-Jaccard et al. 2014; Kruse et al. 2016)
Disease	Special demands for group therapy (Simpson and Reid 2014)
Health sector	Workforce shortage (Jang-Jaccard et al. 2014)
Standards/guidelines	Missing guidelines (Saliba et al. 2012)
Legal framework	Regulatory issues (Hage et al. 2013)
Finance	Lack of funding and reimbursement (Fitzner and Moss 2013; Gros et al. 2013)
Organization	High turnover of medical staff (Jang-Jaccard et al. 2014)
Methodology	Missing proof of cost-effectiveness (Saliba et al. 2012)
Technology	Missing usability (Kruse et al. 2016)

### Telemedicine Community Readiness Model (TCRM)

The TCRM aims to help communities assess their status quo regarding successful telemedicine implementation and scale-up. Based on this initial assessment, improvement measures are proposed, which help to reach higher levels of readiness. The TCRM is based on existing knowledge and approaches (Broens et al. 2007; Cafazzo et al. 2012; Edwards et al. 2000; Ekeland et al. 2012; Goyal et al. 2017; Plested et al. 2006; van Dyk and Schutte 2012). It consists of six levels describing the characteristics of three dimensions. The three dimensions *(status of telemedicine initiatives*, *community involvement*, and *evidence for telemedicine in the community)* and their characteristics are described during their transition from the first level “preplanning” to reaching higher maturity in the sixth, the “professionalization” level (Care4Saxony 2020). Whenever the status quo of a community is defined as being on one of the six levels, improvement measures can be checked for fulfillment and implemented—in case they have not yet been considered. These improvement measures range from providing a step-by-step implementation plan and awareness campaigns about the existence of telemedicine initiatives to ensuring a continuous improvement/performance management and interoperability.

## Method

Selection criteria for our cases are based on purposive or theoretical sampling, which rather seeks to cover the extreme cases instead of representative cases (Gentles et al. 2015). Thus, we decided on cases with maximal extension in technology as well as time, space, and change rate while also considering the availability of historical material. Thus, as case studies, we selected:

1. Sauerbruch, a surgeon at the Charité from 1928 to 1950, who communicated intensively via letters and telephone about the treatment of individual patients and therefore serves as a historical example of teleconsultation using letters, telephone, and telegram as basal technologies (Eckart 2016; Hardinghaus 2019; Sauerbruch 1960).
2. STARPAHC (Space Technology Applied to Rural Papago Advanced Health Care), a project active from 1973 until 1977. It was part of the National Aeronautics and Space Administration (NASA)’s telemedical projects between 1960 and 1990 (Freiburger et al. 2007; Simpson 2013) and is an example of the use of space-suitable technology to overcome very long distances.
3. The use of telemedicine during previous Severe Acute Respiratory Syndrome (SARS), Middle East Respiratory Syndrome (MERS), and Ebola outbreaks (between 2000 and 2016) as an example of telemedicine use in pandemic situations (Chang et al. 2004; Keshvardoost et al. 2020; Lee et al. 2015; Ohannessian 2015) within various healthcare and technology settings, mainly situated in developing countries.

The historical documents of these case studies included project reports, biographies, and evaluation studies (and can be provided upon request).

### Sauerbruch

In the biographies of Sauerbruch, a case was described, where he was involved in treating the English King George V, in 1929. Due to political reasons and historical circumstances, it was not acceptable for an English king to be officially treated by a foreign medical expert. Thus, Sauerbruch could not travel to England to treat him (Hardinghaus 2019; Parth 1970; Sauerbruch 1960). Consequently, he acted as a remote expert (in cooperation with a physician on-site) on treating ailments of the thorax, including the remote analysis of X-ray pictures. As such, this process fully qualifies as telemedicine, especially the phenotype “teleconsultation” (Harst et al. 2019a, b).

### STARPAHC

From the reports of the STARPAHC program, it can be deduced that after NASA developed the program for astronauts, they were inclined to test the advanced technology in a real-life setting (Freiburger et al. 2007). Therefore, NASA implemented telemetry and telemonitoring of “vital health functions as well as their surrounding environment” (Bashshur and Shannon 2009), “monitoring of cardiovascular and pulmonary functions (such as respiration and blood pressure)” from 1961–1972 (p. 191), as well as live videoconferencing, and direct lab analysis (p. 192). The Papago Tribe (now the Tohono O’odham Indian Nation), which is located in Arizona at the border to Mexico with very long distances to cities, let alone clinics, seemed ideal for such a test, although this meant that the project was not planned to initiate a long-term telemedical project in the area (Freiburger et al. 2007; Simpson 2013).

All documents were screened and their content was coded according to the success factor and barrier categories already introduced (see Tables 1 and 2). Also, the TCRM was applied by rating its three dimensions and checking the fulfillment of the proposed improvement measures (Care4Saxony 2020). The categories were used as deductive categories according to the qualitative content analysis proposed by Mayring (2014). Two coders checked the historical documents and did the coding individually. Disagreements were discussed between the coders until a consensus was reached. As two of the three cases chosen took place several decades ago, the available data sets referencing to telemedicine usage were quite scarce. Where no explicit referrals were found, the coding was done implicitly.

## Results

The results of the analysis are clustered according to the three research questions:

- RQ1: What are the success factors of historical telemedicine projects?
- RQ2: What are the barriers to the successful implementation of historical telemedicine projects?
- RQ3: What readiness levels were reached by historical telemedicine projects?

- RQ1: Success factors

### Sauerbruch

Reports from the time of Sauerbruch are sparse and exist in a rather anecdotal form of (auto)biographies, which is why the information drawn on success factors is based on implicit coding. As no other way of having King George V treated by an expert such as Sauerbruch could be found, core readiness can safely be assumed. Based on the high need for treatment of the infected lung, patient readiness must have existed, and according to his autobiography, so has Sauerbruch’s willingness to help. Because X-rays could be sent and received safely by Sauerbruch as well as the attending physician in England, the technological readiness of the postal system must have been sufficient, while legal frameworks did not prevent the remote consultation (Sauerbruch 1960).

### STARPAHC

For STARPAHC, core readiness can be seen as a fact, as the remote location of the Tohono O’odham reservation in Arizona effectively rendered conventional means of caregiving as ineffective, and the Papago people as well as the Indian Health Service (IHS) all supported the novel means of care delivery (Bashshur and Shannon 2009; Freiburger et al. 2007; Simpson 2013). The implemented program was developed by NASA before and was only tested with the Papago people. Therefore, strategic and organizational readiness were provided externally by NASA. Both NASA and IHS went through great length to explain and advertise the benefits of teleconsultation for the people in the reservation and also secured funding for as long as the project ran (Bashshur and Shannon 2009), i.e., financial readiness was achieved. Medical staff in the towns where the STARPAHC sites were located (e.g., Sells and Santa Rosa) was equipped with the technology necessary to perform remote assessments (e.g., high-resolution cameras, Freiburger et al. 2007) and was sufficiently trained to use this technology effectively (Bashshur and Shannon 2009).

### Pandemics

During pandemics such as SARS, MERS, and Ebola, core readiness is achieved mainly through a society-wide understanding that exposing patients as well as health care providers to a contagious viral disease poses great health threats and can lead to an overload of the health care system (Ohannessian 2015). This guarantees the readiness of the health sector as a whole, which is not given in the two previous cases where only isolated projects were studied. Regulatory frameworks within the sector as well as within single institutions are adapted fast to allow for remote consultation, prescription of medication and patient monitoring (Lee et al. 2015). Along with these regulatory changes, legal concerns are ignored (Bokolo 2020; Lee et al. 2015), which allows for legal readiness but cannot be sustained in the long term.

All success factors present in the three cases are summarized in Table 3.

**Table 3:** Success factors in the three historical cases analyzed

	Sauerbruch	STARPAHC	Pandemics
Core readiness	(x)	x	x
Patient readiness	(x)		
Provider readiness	(x)		
Community readiness			
Health sector readiness			x
Strategic readiness		x	
Organizational readiness		x	x
Financial readiness		x	
Legal readiness	(x)		x
Technological readiness	(x)	x	

x = explicitly mentioned in the material; (x) = implicit coding based on the information in the material; blank cell = no information in the material

- RQ2: Barriers

### Sauerbruch

In the material regarding the Sauerbruch example, no barriers could be found. This is because the remote consultation of King George V and his on-site physician was an isolated, short-term process with no intention toward prolongation. As the process was triggered by a clear need and the readiness of all persons involved, no barriers arose over the short period of the consultation (Hardinghaus 2019; Sauerbruch 1960).

### STARPAHC

In the STARPAHC project, health care providers involved found the care provision via digital devices too costly and time-consuming, i.e., the providers and financial issues functioned as a barrier. The withdrawal from the project of the Department of Health, Education, and Welfare, paired with their meager financial contribution to begin with, led to an understaffing of remote sites and therefore marks a clear health sector-related barrier. Also, high concerns regarding the short duration of the project created an organizational barrier, while technical failures during the project’s early stages represented technological barriers (Bashshur and Shannon 2009).

### Pandemics

During pandemics, patients become a barrier when they are not sufficiently trained to use the telemedicine equipment and distrust the technology due to privacy concerns (Bokolo 2020). Despite a non-disputable core readiness triggered by a need to provide sufficient health care without endangering patients, the health sector with its regulations and processes can still be a barrier, mostly when it comes to financing telemedicine equipment and reimbursing the use of telemedicine (Bokolo 2020). Especially in developing countries, rules and legislation for telemedicine use are lacking (Keshvardoost et al. 2020), i.e., the general legal barriers also remain during a pandemic situation. Even though “barriers to inter-jurisdictional telemedicine practice” (Bokolo 2020) are sometimes removed for practitioners during the pandemic, this is not a long-term solution and rather creates additional barriers regarding data security. Missing financial support, especially for low-income earners (Bokolo 2020), or lacking infrastructure, mainly in developing countries (Bokolo 2020; Keshvardoost et al. 2020), also remain during a pandemic and are even more severe as the need for telemedicine is higher during this time.

To summarize the findings, Table 4 illustrates the barriers found in all three cases.

**Table 4:** Barriers in the three historical cases analyzed

Barrier category	Sauerbruch	STARPAHC	Pandemics
Patient			x
Health care provider		x	
Culture			
Disease			
Health sector		x	x
Standards/ guidelines			
Legal framework			x
Finance		x	x
Organization		x	
Methodology			
Technology		x	x

x = explicitly mentioned in the material; blank cell = no information in the material

- RQ3: Readiness levels

### Sauerbruch

Sauerbruch’s consultations can be seen as a small-scale pilot developed by practitioners, while individual members of a community used the telemedicine solution. Evidence was not gained explicitly during the trial but the solution seemed to work well. Therefore, this case can be assessed as having been on level 1 of the TCRM, the level of preplanning, where the planning of first evaluation studies would have been a necessary next step. When considering the measures taken to improve community readiness for telemedicine in the three scenarios, the case of Sauerbruch does not allow for classification as it was a one-time project with no intention of scaling it up any further.

### STARPAHC

STARPAHC serves as an example for a centrally (by the IHS) coordinated small-scale telemedicine pilot, as it enclosed only the Tohono O’odham reservation in Arizona. Between 30% and 41% of the community used the telemedicine solutions provided and an evaluation concept did exist. However, this was not carried out during the testing period (Bashshur and Shannon 2009). Overall, this case can be assessed as having been on level 2, the level of preparation, of the TCRM already.

All relevant actors of the reservation were involved in the basic design of the project (Lovett and Bashshur 1979). However, they were not involved in the following development of the space technology or a follow-up project for their community. Even though basic technical/infrastructural requirements were clear, the technological infrastructure was sufficiently provided, and written contracts existed on all arrangements made during the project (Freiburger et al. 2007), the sudden termination of the project after NASA’s dropout demonstrates a lack of a holistic objective in the community. Further measures taken to support a successful telemedicine implementation were transparent information management to all stakeholders and the provision of training for people supporting telemedicine use and the project itself. NASA could also rely on existing guidelines for decision making in the community, which supported the community climate and were in line with the beliefs of the community, which additionally supported the project’s implementation (Bashshur and Shannon 2009).

### Pandemics

In pandemic situations, large-scale telemedicine initiatives are officially developed by community administrations (Bokolo 2020) and usage by a given community is higher by necessity; however, support varies over age groups and corresponding technological savviness (Bokolo 2020). As we analyzed more than one pandemic in this use case, no assessment of the overall community readiness in pandemic situations can be made.

Generally, in pandemic situations, communities opt for telemedicine solutions bearing in mind the holistic objective to reduce the number of infections and to prevent the health care system from collapse (Lee et al. 2015; Ohannessian 2015). As existing infrastructure is primarily used, concise knowledge of basic infrastructural requirements for telemedicine applications can be gained by those planning their roll-out (Bokolo 2020). Because the safety of health care providers and patients, especially those mainly vulnerable to infection (Keshvardoost et al. 2020)(e.g., elderly and people with chronic conditions), is one of the overarching aims of telemedicine use in pandemic times, recommendations for ethical practice exist (Bokolo 2020). There is, however, little time to involve all stakeholders in the decision-making process and document contractual arrangements, which can serve as a barrier for long-term use of the emergency measures (Lee et al. 2015). Risk management strategies in case of technology malfunctioning or data security breaches are often lacking (Bokolo 2020), and not all regulatory requirements can be followed to the letter (Lee et al. 2015).

## Discussion

The results indicate that especially core readiness is a necessary prerequisite to telemedical projects and it seems to be easily achieved in projects without alternatives for face-to-face medical treatment. This was the case when the King of England needed specialized treatment 70 years ago, and it remains the case when in-person visits threaten the health of both patients and providers due to the risk of contracting a dangerous virus. Furthermore, core readiness appears to entail organizational readiness, as the implementation of remote screening systems showed in 50 health care systems in the U.S. during the first wave of COVID-19 (Hollander and Carr 2020). The rapid provision of guidelines on how to treat patients remotely in pandemic times is a great organizational challenge managed well by many healthcare organizations (Bokolo 2020).

In theory, any form of maturity can be achieved rather quickly in cases of emergency-like pandemics. Also, in pandemic times, barriers (such as data security) long assumed to be paramount can be overcome with sufficient financial, organizational, and technological resources, as Bashshur et al. (2020) argue in a recent collection of lessons learned from the COVID-19 pandemic. However, barriers persist despite core readiness being present, as the STARPAHC project demonstrates: In remote and thinly populated areas, remote consultation of patients is certainly useful; however, funding for any telemedicine system must be ensured beyond the pilot project phase and sustained even when a major stakeholder drops out of the project (Huang et al. 2017; Otto and Harst 2019). Lacking strategic readiness leads to chaotic telemedicine implementation processes during a pandemic, as the COVID-19 pandemic has demonstrated as well (Patel et al. 2020).

This makes clear that a differentiation of the term “barrier” is necessary. While, from the perspective of developers and project planners, barriers might be viewed as factors that prevent the implementation of an innovation and therefore need to be overcome, this might be different from the perspective of the potential end-user of any healthcare technology. From their point of view, these barriers may also represent a protection shield against perceived drawbacks of technology, as depicted in the pandemic use case. For example, patients and providers might fear losing touch with each other (Colorafi et al. 2018). This indicates that digital health applications should never be the only means of health care delivery but rather need to be integrated with other analogous communication channels (Alberts et al. 2011). Additionally, existing theoretical models of acceptance and projects of digital health implementation need to address these above-mentioned fears as well as users’ needs (Harst et al. 2019b). Telemedicine developers should find ways to cope with the barriers instead of ignoring or removing them (Blandford et al. 2020).

From a methodical perspective, our paper demonstrates the possibility to apply the systematics of influencing factors and maturity models derived from modern cases of telemedicine to historic ones. Some limitations, however, remain: The coding revealed that definitions, which were developed against a background of modern technologies, might not always be easily applicable for historical communication channels, such as telegram, telephone or letters, or historical conditions, such as legal regulations, the political context or transportation possibilities. Finally, the historical material we analyzed did not always hold all the information necessary to judge all the factors of readiness, nor all the barriers gained from prior research.

### Implications for current and future projects

It is no coincidence that all three historical use cases focus on teleconsultation as a phenotype of telemedicine (Harst et al. 2019a, b). As the interaction between doctor and patient is the central element in health care delivery (and not the implementation of technology), involving both in the implementation process can leverage one of the most promising potentials of telemedicine use, i.e., tailoring of interventions to individual end-user needs (Holmen et al. 2017). In other words, in not doing so, this potential is lost. Using existing and already commonly used technology (such as letters or telephone in Sauerbruch’s times) or established processes of decision-making (such as the two-step flow with opinion leaders in indigenous communities) might be more useful than the implementation of totally new ones. Thus, considering the patient and provider perspectives in a participatory development process might help overcome the notion that the proliferation of technology alone means it fits with contextual requirements (Esser and Goossens 2009). Therefore, a holistic assessment of the environment into which the technology is to be implemented is more important than the latest technological development (Hastall et al. 2017). Consequently, implementation strategies, soundly based on adequate methods of user participation, are the most pressing future research needs in the field of telemedicine (Timpel and Harst 2020).

The Sauerbruch case and pandemic cases revealed that core needs can be temporarily so high that they can overrule privacy considerations or concerns of limited understanding (e.g., due to missing nonverbal cues or the possibility to exam the whole body) for a certain amount of time. However, so far, learnings from previous pandemics such as SARS and MERS have not helped to sustain teleconsultation systems (e.g., Blandford et al. 2020). This might be because some barriers (or concerns) of telemedicine, such as those mentioned above, will persist, no matter how far the technology is developed.

Therefore, current guidelines, e.g., for the treatment of diabetes, suggest two important extensions for telemedicine projects. First, they suggest incorporating the technology as a supplement into a broad set of measures for diabetes self-management and support, instead of seeing it as an alternative to usual care (Haas et al. 2014; Schwarz et al. 2018). Second, they suggest extending the set of relevant outcomes that are looked at. This means, that in addition to medical parameters (which are mostly used as indicators of effectiveness), project evaluations should also consider the satisfaction with the doctor–patient relationship, such as trust, and process-related indicators, such as time-savings in daily practice (NICE 2019).

## Conclusion

On a meta-level, we see that a historical perspective allows for more general conclusions above the details of a specific technology or disease-specific medical needs while results of the current research landscape are confirmed. This also applies for, e.g., the importance of core readiness, which is a construct that is derived from rather modern research. Our analysis indicates that core readiness (i.e., needs and dissatisfaction with the status quo) plays a central role for telemedicine and has to be a prerequisite for future projects in research as well as in practice. In addition, evaluations should include the perceived fulfillment of such basic needs. We can also see this when we look at the broad spectrum of current examples of telemedicine, such as healthcare provision in remote areas in Australia (The University of Queensland 2020), the use of telemedicine in the recent COVID-19 pandemic (Keshvardoost et al. 2020) or up-and-running teleconsultation networks, e.g., emergency stroke units (Uniklinikum Carl Gustav Carus 2017).

Beyond these case studies and looking at similar projects in the long history of telemedicine, we can conclude from our analysis that problems in health care delivery have in all times stimulated innovative technological and communicational means, and strategies to deliver health care services over distance (Sood et al. 2007). This also implies that the relationship between medical professionals and their patients needs communication channels for trustful exchange to achieve successful disease management and curing processes.

At the same time, we can also learn a lot from short-term or so-called failed projects. A sudden change of circumstances, such as the use of telemedicine during pandemics, can tell us plenty about which barriers are persistent and which can be overcome by political, financial, legal, or technological developments, e.g., to learn which areas still lack net coverage or which privacy regulations are still necessary (Bokolo 2020). It can be concluded for future research and practice projects that it is important to strategically include an analysis of barriers and their changeability. Thus, from a long-term perspective, the sensitive dealing with barriers might be rather viewed as the most important success factor and the readiness to do so expresses a maturity level on its own.
